# Radiomic Cancer Hallmarks to Identify High-Risk Patients in Non-Metastatic Colon Cancer

**DOI:** 10.3390/cancers14143438

**Published:** 2022-07-15

**Authors:** Damiano Caruso, Michela Polici, Marta Zerunian, Antonella Del Gaudio, Emanuela Parri, Maria Agostina Giallorenzi, Domenico De Santis, Giulia Tarantino, Mariarita Tarallo, Filippo Maria Dentice di Accadia, Elsa Iannicelli, Giovanni Maria Garbarino, Giulia Canali, Paolo Mercantini, Enrico Fiori, Andrea Laghi

**Affiliations:** 1 Radiology Unit, Department of Medical Surgical Sciences and Translational Medicine, Sapienza University of Rome-Sant’Andrea University Hospital, Via di Grottarossa, 1035-1039, 00189 Rome, Italy; michela.polici@uniroma1.it (M.P.); marta.zerunian@uniroma1.it (M.Z.); antonella.delgaudio@uniroma1.it (A.D.G.); emanuela.parri211990@gmail.com (E.P.); mariaagostinag@gmail.com (M.A.G.); domenico.desantis@uniroma1.it (D.D.S.); elsa.iannicelli@uniroma1.it (E.I.); andrea.laghi@uniroma1.it (A.L.); 2 Surgery Unit, Department of Medical Surgical Sciences and Translational Medicine, Sapienza University of Rome-Sant’Andrea University Hospital, Via di Grottarossa, 1035-1039, 00189 Rome, Italy; giulia.tarantino@hotmail.com (G.T.); giovannimaria.garbarino@uniroma1.it (G.M.G.); giulia.canali@uniroma1.it (G.C.); paolo.mercantini@uniroma1.it (P.M.); 3 Department of Surgery “Pietro Valdoni”, Sapienza University of Rome, 00161 Rome, Italy; mariarita.tarallo@uniroma1.it (M.T.); filippomaria.denticediaccadia@uniroma1.it (F.M.D.d.A.); enrico.fiori@uniroma1.it (E.F.)

**Keywords:** radiomics, colon cancer, cancer hallmarks, risk prediction

## Abstract

**Simple Summary:**

Colon cancer is one of the most common cancers in the world, and the therapeutic workflow is dependent on the TNM staging system and the presence of clinical risk factors. However, in the case of patients with non-metastatic disease, evaluating the benefit of adjuvant chemotherapy is a clinical challenge. Radiomics could be seen as a non-invasive novel imaging biomarker able to outline tumor phenotype and to predict patient prognosis by analyzing preoperative medical images. Radiomics might provide decisional support for oncologists with the goal to reduce the number of arbitrary decisions in the emerging era of personalized medicine. To date, much evidence highlights the strengths of radiomics in cancer workup, but several aspects limit the use of radiomics methods as routine.

**Abstract:**

The study was aimed to develop a radiomic model able to identify high-risk colon cancer by analyzing pre-operative CT scans. The study population comprised 148 patients: 108 with non-metastatic colon cancer were retrospectively enrolled from January 2015 to June 2020, and 40 patients were used as the external validation cohort. The population was divided into two groups—High-risk and No-risk—following the presence of at least one high-risk clinical factor. All patients had baseline CT scans, and 3D cancer segmentation was performed on the portal phase by two expert radiologists using open-source software (3DSlicer v4.10.2). Among the 107 radiomic features extracted, stable features were selected to evaluate the inter-class correlation (ICC) (cut-off ICC > 0.8). Stable features were compared between the two groups (T-test or Mann–Whitney), and the significant features were selected for univariate and multivariate logistic regression to build a predictive radiomic model. The radiomic model was then validated with an external cohort. In total, 58/108 were classified as High-risk and 50/108 as No-risk. A total of 35 radiomic features were stable (0.81 ≤ ICC <  0.92). Among these, 28 features were significantly different between the two groups (*p* < 0.05), and only 9 features were selected to build the radiomic model. The radiomic model yielded an AUC of 0.73 in the internal cohort and 0.75 in the external cohort. In conclusion, the radiomic model could be seen as a performant, non-invasive imaging tool to properly stratify colon cancers with high-risk disease.

## 1. Introduction

Colon cancer is the fifth-most-common cancer in terms of incidence and mortality, with 1,480,000 new cases in 2020 worldwide [1]. The main therapeutic options are surgical resection and adjuvant chemotherapy in non-metastatic colon cancer; however, the evaluation of the overall adjuvant chemotherapy benefit in patients with a high risk of recurrence is a clinical challenge [2]. The decision is based on the TNM staging system [3], which represents the most important parameter; colon cancer patients at stage III are globally recognized as patients who can benefit from chemotherapy, while for those at stage II with other clinical risk factors, the advantages of chemotherapy are still debated [2,4]. In presence of clinical risk factors, the final strategy is often arbitrarily decided by the oncologist. Nevertheless, much evidence has revealed that not all clinical risk features are equal, not all affect overall survival, and the decision to treat colon cancer with adjuvant chemotherapy should be assessed in a multidisciplinary approach [5].

In this context, radiomics could play a pivotal role in colon cancer workup with the expectancy to help clinicians in identifying patients with high-risk disease. Radiomics might be used as a non-invasive imaging biomarker and be able to provide a quantitative evaluation of medical images, with the chance to shift the imaging approach from conventional, which is qualitative and subjective, to quantitative. This new field of imaging has the ability to extract a large amount of data from specific regions of interest (ROIs), including differences in image texture, spatial resolution, and pixel interrelations, which are rather imperceptible to the human eye, in order to quantitatively outline image phenotypic characteristics at an ultrastructural level [6,7]. To date, the radiomics approach has been extensively investigated in cancer patients with a specific focus on tumor diagnosis, staging, prognosis prediction, and long-term monitoring [6,8,9,10].

Concerning colorectal cancer, several managerial aspects were explored with the aim of testing the performance of radiomics as an additional tool in a clinical setting. In particular, the main fields examined were the preoperative assessment of the mutational panel, the differentiation between low- and high-grade colon cancer, and the prediction of nodal metastases [11,12,13,14,15,16,17]. Almost all studies were performed on baseline CT scans by outlining the primary tumor; overall, the results achieved good and consistent efficiency, especially in mutational paneling and in identifying high-risk clinical factors, reinforcing the idea that radiomics could play a central role in colon cancer patient workup. Nevertheless, radiomics has numerous shortcomings that make daily use extremely difficult. Among these, the lack of standardization and validation, poor reproducibility, and missing prospective multicentric studies represent the main drawbacks that must be overcome to introduce the radiomics approach to the clinical routine [6].

To the best of our knowledge, no studies have assessed the performance of radiomics in stratifying patients with high-risk disease in patients with non-metastatic colon cancer. We built and validated a radiomic model with the purpose of preoperatively identifying patients with high-risk colon cancer who could benefit from adjuvant chemotherapy.

## 2. Materials and Methods

### 2.1. Patient Selection

**Figure 1:**
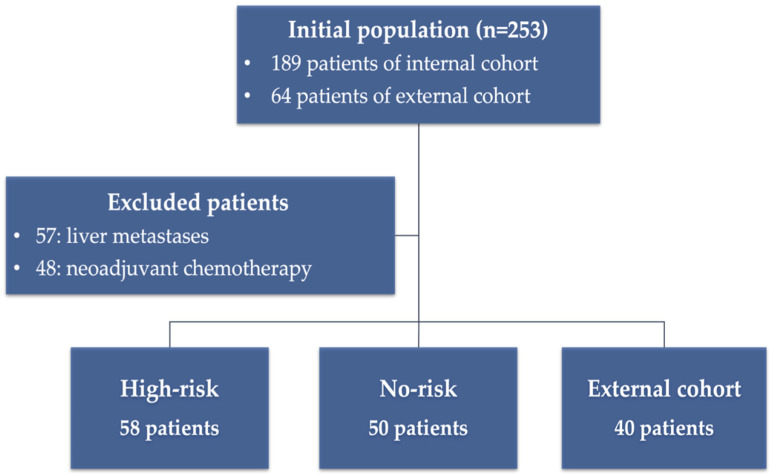
Patient recruitment flow-chart.

This retrospective observational study was conducted in accordance with the Declaration of Helsinki, and it was approved by the ethical committee of Sant’Andrea University Hospital (ref. nr. CE 6597/2021). In total, 253 patients (189 internal cohort and 64 external cohort) with new diagnoses of non-metastatic colon cancer from January 2015 to June 2020 were enrolled, and all patients provided informed consent. For each patient, we collected epidemiological and clinical data, including their age, sex, perineural invasion (PNI), lymphovascular invasion (LVI), budding, staging, tumor location, and microsatellite instability status. The population was selected in accordance with the following inclusion criteria: (I) radical surgery, (II) availability of clinical and histological data, (III) availability of portal phase on the baseline CT scan, and having (IV) stage I, II, or III. Exclusion criteria: (I) stage IV, (II) patients previously treated with neoadjuvant chemotherapy, and (III) patients with advanced colon adenomas. The internal cohort was divided into High-risk and No-risk according to the presence of at least one of the following risk factors: staging T4, LVI, PNI, budding, and nodal metastases [2] (Figure 1). An external validation cohort of 40 non metastatic colon cancer (27 male and 13 female) was selected following the same inclusion and exclusion criteria described for the internal cohort. External cohort was used to test the predictive models.

### 2.2. CT Acquisition Protocol

All patients were studied with contrast-enhanced CT scans by using 128-slice CT (GE Revolution EVO Slice CT Scanner, GE Healthcare, Milwaukee, WI, USA) before surgery. The CT scans were acquired with the patients in supine position and performed at end-inspiration in the cranio–caudal direction—the Z-axis was set covering the entire abdomen.

The contrast medium (CM) volume was tailored for each patient following the lean body weight [18,19]:

$$\mathrm{CM}\;\mathrm{volume}\left(\mathrm{mL}\right)=\frac{0.7\mathrm{gI}\times \mathrm{LBW}\left(\mathrm{kg}\right)}{\mathrm{CM}\;\mathrm{concentration}\left(\frac{\mathrm{mgI}}{\mathrm{mL}}\right)}$$

The bolus of contrast medium (Iodixanolo 320 mg I/mL, Visipaque 320; GE Healthcare, Milwaukee, WI, USA) and the subsequent saline solution (50 mL) were injected using a contrast media injection system (MEDRAD^®^ Centargo CT Injection System, version 1.4.0, Bayer AG, Berlin, Germany) with the flow rate fixed at 3.5 mL/s through antecubital venous access (18–20 gauge). The bolus-tracking method (Smart Prep, GE, Milwaukee, WI, USA) was used for the multiphase CT scan acquisition by setting a 100 HU-threshold region of interest at the tripod celiac level within the abdominal aorta. For each patient, the unenhanced, late arterial (18 s from the threshold), and portal venous (70 s from the threshold achieved) phases were performed. The following CT technical specifications were set: tube voltage 100 kV, spiral pitch factor 0.98, tube current modulation 130–300 mAs by using SMART mA (GE Healthcare, Milwaukee, WI, USA), time of rotation 0.6 s, and collimation 64 × 0.625 mm.

### 2.3. CT Scans Segmentation Analysis

**Figure 2:**
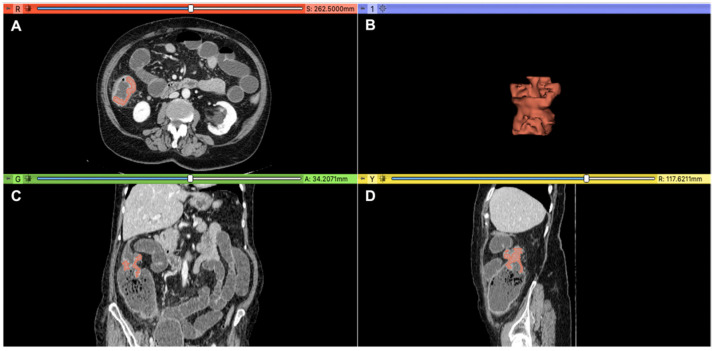
Colon cancer 3D segmentation in the portal phase performed by using Slicer software (version 4.10.2, https://download.slicer.org, accessed on 17 March 2021). Panel (**A**) displays the axial, (**B**) 3D volumetric segmentation, (**C**) coronal, and (**D**) sagittal plans.

All colon cancers were segmented by two expert abdominal radiologists (E.I. and D.C., with 25 and 10 years of experience, respectively), who independently performed a volumetric segmentation of colon cancer on the preoperative CT scans at the portal phase. The open-source 3D Slicer software (version 4.10.2, https://download.slicer.org, accessed on 17 March 2021) was used for segmentation. The volumetric region of interest was manually outlined slice-by-slice in order to cover the entire colon cancer volume and avoid including the surrounding pericolic fat and healthy large bowel wall in the segmentation (Figure 2).

### 2.4. Radiomics Extraction

To extract 107 radiomic features from the CT portal venous phase, the 3D Slicer Radiomics extension (pyradiomics library [20]) was used. The 107 features extracted included: First-Order statistics, 19 features; 2D and 3D Shape, 13 features; Neighboring Gray Tone Difference Matrix (NGTDM), 5 features; Gray Level Size Zone Matrix (GLSZM), 16 features; Gray Level Co-Occurrence Matrix (GLCM), 24 features; Gray Level Dependence Matrix (GLDM), 14 features; and Gray Level Run Length Matrix (GLRLM), 16 features.

### 2.5. Statistical Analysis

All continuous data were evaluated as the mean ± standard deviation. The interobserver variability, evaluating the inter-class correlation (ICC), was used to select the stable radiomic features, and radiomic features achieving ICC > 0.8 were maintained for the next statistical analysis steps [21]. Student’s *t*-test and the Mann–Whitney U test were used for the comparison of the continuous variables of High-risk and No-risk patients according to Gaussian normality or non-normality, respectively. Univariate enter logistic regression was used to test stable radiomic features (ICC > 0.8) as predictors of high-risk cancer. All significant (*p* < 0.05) parameters were selected for the multivariable enter logistic regression analysis with the goal to build a radiomic model to predict High-risk colon cancer. The predictive radiomic model, validated through the external cohort, was classified as Type 3 according to the transparent reporting of a multivariable prediction model for individual prognosis or diagnosis (TRIPOD) statements [22]. Statistical significance was considered at *p* < 0.05. Statistical analysis was conducted using MedCalc (MedCalc Software, version15, Ostend, Belgium).

## 3. Results

### 3.1. Study Population

In total, 108 patients from the internal population matched the inclusion and exclusion criteria (median age 72, Male 56/108); 58 patients were stratified as High-risk and 50 as No-risk. In the-sub analysis of the High-risk patients, concerning T staging, 1 (1.7%) was T1, 3 (5.2%) were T2, 33 (56.9%) were T3, 17 (29.3%) were T4a, and 4 (6.9%) were T4b. Regarding the presence of risk factors, 36 (62%) were LVI-positive, 4 (6.9%) were PNI-positive, 34 (58.6%) were budding-positive, and 16 (27.6%) were N-positive. In the sub-analysis of the No-risk patients, concerning T staging, 1 (2%) was T1, 8 (16%) was T2, and 41 (82%) were T3 (Table 1). Concerning the external validation cohort, 40 patients were selected (27 male and 13 female).

### 3.2. Feature Selection and Radiomic Analysis

In total, 107 radiomic features were extracted from the 3D segments of colon cancer in the portal phase of the baseline CT scans. The analysis of ICC revealed that only 35 radiomic features (8 Shape, 5 First order, 3 GLCM, 5 GLDM, 6 GLRLM, 7 GLSZM, and 1 NGTDM feature) were stable (0.81  ≤  ICC  <  0.92). Among the stable features, 28 features (7 Shape, 3 First order, 1 GLCM, 4 GLDM, 6 GLRLM, 6 GLSZM, and 1 NGTDM feature) were significantly different between the High-risk and No-risk patients (0.004 ≤ *p* < 0.05) (Table 2).

### 3.3. Univariate and Multivariate Analyses

**Figure 3:**
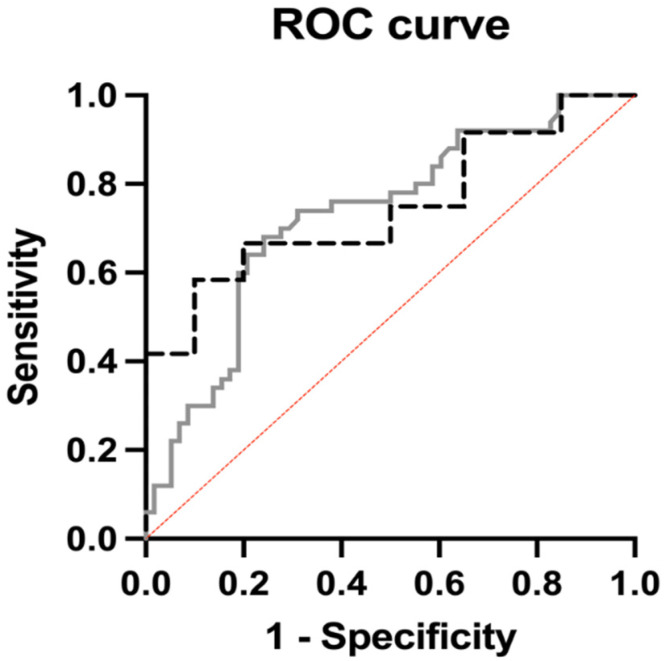
Performance of radiomic model to identify high-risk colon cancer in the internal (dotted black line) and external (solid gray line) cohorts, reaching AUC of 0.73 and 0.75, respectively.

All significant stable radiomic features were tested by using univariable logistic regression analysis to evaluate the correlation with high-risk colon cancer. Univariate analysis showed that nine radiomic features (one First-Order, one GLCM, five GLRLM, and two GLSZM features) were significantly associated with high-risk cancer, with the *p* values ranging from 0.01 to 0.05 and OR 1. Among these features, one Shape (SurfaceVolumeRatio), three GLRLM (RunLengthNonUniformityNormalized, RunPercentage, and ShortRunEmphasis), and one GLSZM (ZonePercentage) were predictors of high-risk cancer, with *p* values ranging from 0.01 to 0.05 and OR between 13.6 and 157 × 104. Meanwhile, one GLCM (Idmn), two GLRLM (LongRunEmphasis and RunVariance), and one GLSZM (SmallAreaEmphasis) showed an inverse correlation with high-risk cancer, with a *p* value of 0.01 to 0.02 and OR between 0.84 and 4.2004 × 10^−17^. The remanent stable radiomic features showed no significant correlation with high-risk cancer or indifferent values of OR. Multivariate analysis was conducted to build the radiomic model by including the radiomic features with a significant correlation with high-risk cancer. The radiomic model showed good performance, with AUC of 0.73 (95% CI, 0.63–0.82; *p* < 0.001), positive predictive power of 71.43%, and negative predictive power of 69.7%. The results were validated through the external cohort, in which the radiomic model yielded an AUC of 0.75 (95% CI, 0.55–0.94; *p* = 0.02), with positive predictive power of 70% and negative predictive power of 77.3% (Figure 3 and Table 3).

## 4. Discussion

In this study, we developed a radiomic model to predict high-risk disease in non-metastatic colon cancer by performing a volumetric segmentation of primary tumors on baseline CT scans. All patients were treated with surgical resection, and we considered clinicopathological data as a reference standard to divide the starting population into High-risk and No-risk patients according to the presence of at least one clinical risk factor, such as staging T4, LVI, PNI, budding, and nodal metastases [5,23]. We analyzed all pre-operative CT scans in the portal phase, extracting from each volumetric tumor segmentation multiple radiomic features that were reduced according to the value of ICC, to maintain only the stable features. Then, the stable radiomic features were compared by testing the differences between high-risk and no-risk patients, and the significant radiomic features were used to build a radiomic predictive model. The model achieved good performance in predicting high-risk disease with an AUC of 0.73, highlighting the promising role of radiomics in patient risk stratification. It was also validated through an external cohort, in which the AUC was confirmed good, yielding a value of 0.75.

To date, radiomics have been widely described as a new field of quantitative imaging, having the ability to outline the micro-architecture and heterogeneity of the tissues through a large volume of numeric data extracted from medical images [6]. These high-dimensional data could be an expression of tumor aggressiveness, with the possible opportunity to overcome the limitations of conventional imaging, which is subjective and qualitative [24,25]. Focusing on colon cancer, conventional imaging has consistent limitations in identifying the main high-risk clinical factors, such as nodal metastases, LVI, and PNI. Among these, nodal involvement was the factor most commonly investigated by using conventional imaging, and no consistent results have been obtained. In fact, almost all qualitative evaluations to predict the risk of nodal metastases were found to be non-performing [26].

In this context, radiomics could be seen as a novel tool to stratify patients affected by colon cancer by providing some additional quantitative data, with the goal of outlining the tumor phenotype and predicting patient prognosis before starting the therapeutic workflow. Recently, the group of Yao X. [27] demonstrated an opportunity to use a radiomics approach to predict disease-free survival in colon cancer patients. They compared the predictive value of the TNM staging system, clinical model, and radiomics. The radiomics signature was proven to be more efficient than TNM and the clinical model in predicting the patient’s prognosis. Similar results were reported by Dai W. et al., who tested radiomics as an imaging biomarker to identify patients with poor prognosis. They evaluated the potentiality of a quantitative approach to assess overall survival and relapse-free survival by analyzing preoperative CT scans. The authors obtained good performance for both endpoints, reaching AUCs of 0.77 and 0.74 in predicting the overall survival and relapse-free survival, respectively. These studies enhanced the potential value of radiomics as an imaging biomarker in non-metastatic colon cancer that will help clinicians to choose the best treatment option according to patient risk stratification.

Currently, all colon cancers at stages III and II with high-risk clinical features are recommended to be treated with adjuvant chemotherapy. However, the benefits of adjuvant chemotherapy in stage II with high-risk clinical features are debated, mainly due to the conflicting results of some clinical studies [28,29]. The option of adjuvant chemotherapy in high-risk colon cancer at stage II is still arbitrary and often guided by subjective evaluation by oncologists. In such a scenario, we decided to use clinicopathological data only to stratify the patients into High-risk and No-risk groups and to only test the performance of the radiomic model. The study design was weighted on the basis of the controversial results present in the literature regarding the combined model, clinical–radiomics, to preoperatively identify colon cancer at stage III. On the one hand, a recent study stated that a clinical–radiomics nomogram was superior in the preoperative prediction of nodal metastases [30]. Conversely, in a different study, it was reported that the radiomics signature achieved the best performance in N staging in comparison with the combined model [13]. These opposite results guided our decision to only consider the histological data to stratify the patients. We wanted to avoid any confounding results concerning clinical data, even considering that our main investigation aim was to look at the radiomics approach as a supporting tool for clinicians without any possibilities to replace the clinical approach. Nevertheless, we did not include the stratification of patients with several novel biomarkers concerning the mutational panel (e.g., BRAF, KRAS, and microsatellite instability) [2,4]. MSI was evaluated, and 10.3% and 20% of patients exhibited MSI among the High-risk and No-risk groups, respectively. MSI status is an important prognostic factor to outline therapeutic management for patients; it has been shown that MSI is associated with a reduced risk of metastatic disease in stages I and II colon cancer [31]. However, in colon cancer at stages III and IV, MSI is a worse prognostic factor—these patients are less responsive to conventional chemotherapy and need to be treated with target therapy, such as Pembrolizumab [32]. Following these controversial data in stages I/II and III/IV, we decided to not evaluate the presence of MSI as a prognostic factor. The remanent paneling of the mutational status has not been widely used as routine in colon cancer, especially in previous years, and this information was not available at the moment of analysis, also considering the retrospective nature of the study.

In the new era of personalized medicine, quantitative imaging could be central in the management of colon cancer by providing clinicians with a non-invasive imaging biomarker to properly tailor therapy, especially in doubtful cases. The number of arbitrary decisions should be reduced, and a structured workflow is required to ensure a therapeutic program tailored to each patient. Radiomics could be seen as a quantitative tool to guide clinicians and to limit the over- and under-treating of patients who may or may not benefit from chemotherapy. Radiomics could be also considered as an objective imaging biomarker to monitor oncologic patients during follow-up, also by quantifying the ultrastructural changes, especially in the case of metastatic disease [6,8,9,10]. Despite the high potential of radiomic analysis in a pre-operative clinical setting, the real strengths in predicting patient outcomes have been verified; however, the leading limitations include poor standardization, low reproducibility of results, and different acquisition parameters between different centers [6]. In fact, between the various cancer-research centers, there is a disparity concerning several factors inherent to the CT acquisition workflow, such as contrast-enhanced CT phases, iterative reconstructions, and the total volume of contrast medium, which could affect the consistency of radiomics [33].

This study has several limitations; firstly, the retrospective nature of the study; secondly, the small samples of internal and external cohorts; thirdly, data of patient outcomes were missing, and survival analysis was not performed; and fourthly, the lack of follow-up data. In the future, these limitations could be overcome by performing a second analysis step based on a large prospective enrollment, in which many clinical and survival data (e.g., complete genetic panel and treatment decision) will be collected, and also by following the patients selected in this first retrospective step.

## 5. Conclusions

To sum up, we can conclude that the radiomic model might play a pivotal role in future colon cancer workup, focusing on patient risk stratification in a pre-operative clinical setting. This approach might serve as a supporting tool for clinicians, with the expectancy to enter structured treatment management, allowing a personalized therapeutic strategy to be obtained.

## Figures and Tables

**Table 1:** Patient clinical data.

High Risk (58/108)	N Patients	%	No Risk (50/108)	N Patients	%
**T**			**T**		
○ **T1**	1	1.7	○ **T1**	1	2
○ **T2**	3	5.2	○ **T2**	8	16
○ **T3**	33	56.9	○ **T3**	41/50	82
○ **T4a**	17	29.3	○ **T4a**	0/50	0
○ **T4b**	4	6.9	○ **T4b**	0/50	0
**LVI**			**LVI**		
○ **LVI+**	36/58	62	○ **LVI+**	0/50	-
○ **LVI−**	22/58	38	○ **LVI−**	50/50	100
**PNI**			**PNI**		
○ **PNI+**	4/58	6.9	○ **PNI+**	0/50	-
○ **PNI−**	54/58	93.1	○ **PNI−**	50/50	100
**BUDDING**			**BUDDING**		
○ **Budding+**	34/58	58.6	○ **Budding+**	0/50	-
○ **Budding−**	24/58	41.4	○ **Budding−**	50/50	100
**Nodes**			○ **Nodes**		
○ **N0**	42/58	72.5	○ **N0**	50/50	100
○ **N1a**	4/58	6.9	○ **N1a**	-	-
○ **N1b**	5/58	8.6	○ **N1b**	-	-
○ **N2a**	5/58	8.6	○ **N2a**	-	-
○ **N2b**	2/58	3.4	○ **N2b**	-	-
**MSI**	6/58	10.3	**MSI**	10/50	20
**Tumor location**			**Tumor location**		
○ **Right-sided**	29/58	50	○ **Right-sided**	24/50	48
○ **Trasverse**	3/58	5.2	○ **Trasverse**	3/50	6
○ **Left-sided**	26/59	44.8	○ **Left-sided**	23/50	46

T: T staging; LVI: lymphovascular invasion; PNI: perineural invasion; MSI: microsatellite instability.

**Table 2:** Stable radiomic features in comparison between High-risk and No-risk patients.

Radiomic Features	High Risk	No Risk	ICC	*p*
	**Mean ± SD**	**Mean ± SD**		
**Shape_LeastAxisLength**	23.34 ± 10.43	28.38 ± 12.24	0.82	**0.02**
**Shape_Maximum2DDiameterColumn**	43.20 ± 18.48	56.74 ± 23.58	0.87	**0.003**
**Shape_Maximum2DDiameterSlice**	49.30 ± 19.09	58.63 ± 22.76	0.90	**0.02**
**Shape_MeshVolume**	21,047.06 ± 26,389.25	39,659.83 ± 43,204.46	0.81	**0.02**
**Shape_MinorAxisLength**	31.52 ± 11.29	38.45 ± 13.76	0.91	**0.004**
**Shape_SurfaceArea**	6507.93 ± 4960.29	10,070.17 ± 7988.04	0.87	**0.02**
**Shape_SurfaceVolumeRatio**	0.46 ± 0.19	0.38 ± 0.16	0.85	**0.02**
**Shape_Maximum3DDiameter**	56.72 ± 22.63	65.57 ± 24.46	0.89	0.07
**First Order_VoxelVolume**	21,532.50 ± 26,508.12	40,253.22 ± 43,386.76	0.91	**0.009**
**First Order_Energy**	5,272,857.19 ± 6,465,846.03	9,614,816.02 ± 10,922,495.83	0.90	**0.03**
**First Order_TotalEnergy**	142,367,144.12 ± 174,577,842.8	259,600,032.54 ± 294,907,387.3	0.86	**0.03**
**First Order_Maximum**	149.91 ± 30.88	147.02 ± 27.59	0.82	0.61
**First Order_Mean**	74.72 ± 15.85	72.08 ± 18.64	0.88	0.81
**GLCM_Idmn**	0.98 ± 0.01	0.98 ± 0.01	0.89	**0.03**
**GLCM_Icm2**	0.29 ± 0.10	0.26 ± 0.09	0.85	0.08
**GLCM_SumAverage**	9.55 ± 6.30	11.28 ± 7.01	0.85	0.16
**GLDM_DependenceNonUniformity**	39.95 ± 42.32	70.22 ± 72.70	0.87	**0.02**
**GLDM_GrayLevelNonUniformity**	439.23 ± 537.16	877.22 ± 985.89	0.86	**0.01**
**GLDM_LargeDependenceEmphasis**	152.75 ± 71.20	185.66 ± 83.91	0.88	**0.02**
**GLDM_SmallDependenceEmphasis**	0.06 ± 0.03	0.05 ± 0.02	0.90	**0.03**
**GLDM_SmallDependenceLowGrayLevelEmphasis**	0.02 ± 0.01	0.01 ± 0.01	0.89	0.06
**GLRLM_GrayLevelNonUniformity**	199.18 ± 202.85	342.55 ± 321.67	0.88	**0.02**
**GLRLM_LongRunEmphasis**	4.34 ± 2.62	5.61 ± 3.63	0.85	**0.03**
**GLRLM_RunLengthNonUniformityNormalized**	0.47 ± 0.09	0.43 ± 0.10	0.81	**0.02**
**GLRLM_RunPercentage**	0.62 ± 0.09	0.58 ± 0.11	0.82	**0.04**
**GLRLM_RunVariance**	1.37 ± 1.10	2.04 ± 1.92	0.87	**0.04**
**GLRLM_ShortRunEmphasis**	0.70 ± 0.07	0.67 ± 0.08	0.87	**0.03**
**GLSZM_ LargeAreaEmphasis**	12,801.55 ± 22,785.03	32,877.79 ± 45,848.79	0.90	**0.006**
**GLSZM_ LargeAreaHighGrayLevelEmphasis**	609,276.79 ± 17,04,878.107	1,908,734.67 ± 4,536,097.03	0.90	**0.01**
**GLSZM_ LargeAreaLowGrayLevelEmphasis**	693.99 ± 1,234.32	1714.36 ± 3566.59	0.82	**0.03**
**GLSZM_SmallAreaEmphasis**	0.58 ± 0.17	0.64 ± 0.11	0.87	**0.04**
**GLSZM_ZonePercentage**	0.07 ± 0.04	0.05 ± 0.04	0.89	**0.01**
**GLSZM_ZoneVariance**	12,224.58 ± 22,231.55	31,666.31 ± 44,755.72	0.91	**0.008**
**GLSZM_ SmallAreaHighGrayLevelEmphasis**	16.35 ± 29.18	23.66 ± 31.59	0.90	0.06
**NGTDM_ Coarseness**	0.04 ± 0.06	0.02 ± 0.04	0.88	**0.01**

SD: Standard deviation; ICC: inter-class correlation; P: *p* value; GLCM: Gray Level co-occurrence matrix; GLDM: Gray Level Dependence Matrix; GLRLM: Gray Level Run Length Matrix; GLSZM: Grey Level Size Zone Matrix; NGTDM: Neighboring Gray Tone Difference Matrix.

**Table 3:** Multivariate logistic regression to test the performance of the radiomic model in predicting high-risk colon cancer in internal and external cohorts.

Radiomic Variable	Internal Cohort Radiomic Model		External Cohort	
	**OR (95% CI)**	**Coefficient**	**OR (95% CI)**	**Coefficient**
**Shape_SurfaceVolumeRatio**	0.79 (7.82 × 10^−22^ to 5.42 × 10^30^)	−0.24	227.1 (6.65 × 10^−5^ to 1,771,984,111)	5.42
**GLCM_Idmn**	3,647,282,668 (2.973 × 10^−10^ to 1.16 × 10^+30^)	22.02	1.21 × 10^+20^ (2.07 × 10^−28^ to 1.05 × 10^+74^)	46.25
**GLRLM_LongRunEmphasis**	0.02 (0.0003 to 1.42)	−3.63	58.36 (0.0004 to 183,464,701)	4.067
**GLRLM_RunLengthNonUniformityNormalized**	5.99 × 10^+14^ (3.38 × 10^−15^ to 1.37 × 10^+44^)	34.03	8.20 × 10^+38^ (9.29 × 10^−60^ to 5.55 × 10^+145^)	89.60
**GLRLM_RunPercentage**	4.7 × 10^+18^ (0.005 to 1.66 × 10^+42^)	42.99	1.54 × 10^−54^ (1.33 × 10^−131^ to 126,781)	−123.9
**GLRLM_RunVariance**	1537 (1.24 to 4,121,443)	7.34	1.89 × 10^−5^ (1 × 10^−17^ to 36,401)	−10.87
**GLRLM_ShortRunEmphasis**	3.54 × 10^−45^ (2.35 × 10^−88^ to 0.0006)	102.4	735,727,550 (1.49 × 10^−77^ to 1.94 × 10^+100^)	20.42
**GLSZM_SmallAreaEmphasis**	38.22 (0.49 to 3684)	3.64	0.89 (9.58 × 10^−6^ to 64,647)	−0.11
**GLSZM_ZonePercentage**	6.87 × 10^−8^ (6.04 × 10^−19^ to 1659)	−16.49	42,583,803 (1.39 × 10^−22^ to 1.75 × 10^+40^)	17.57
***p*** **value**	**<0.0001**		**0.02**	
**AUC**	**0.73**		**0.75**	
**Positive Predictive Power**	**71.4%**		**70%**	
**Negative Predictive Power**	**69.7%**		**77.3%**	

OR: Odds ratio; AUC: Area under curve; GLCM: Gray Level Co-occurrence Matrix; GLRM: Gray Level Run Length Matrix; GLSZM: Grey Level Size Zone Matrix.

## Data Availability

Data supporting the reported results can be obtained from the corresponding author.
